# Effect of Pulsed Light on Quality of Shelled Walnuts

**DOI:** 10.3390/foods11091186

**Published:** 2022-04-19

**Authors:** Vicente Manuel Gómez-López, Luis Noguera-Artiaga, Fernando Figueroa-Morales, Francisco Girón, Ángel Antonio Carbonell-Barrachina, José Antonio Gabaldón, Antonio Jose Pérez-López

**Affiliations:** 1 Catedra Alimentos para la Salud, Campus de los Jerónimos, Universidad Católica San Antonio de Murcia (UCAM), 30107 Murcia, Spain; vmgomez@ucam.edu; 2 Research Group “Food Quality and Safety”, Centro de Investigación e Innovación Agroalimentaria y Agroambiental (CIAGRO-UMH), Miguel Hernández University of Elche (UMH), Carretera de Beniel km 3.2, 03312 Orihuela, Spain; lnoguera@umh.es (L.N.-A.); angel.carbonell@umh.es (Á.A.C.-B.); 3 Department of Food Technology and Nutrition, Catholic University of San Antonio, Campus de los Jerónimos s/n, Guadalupe, 30107 Murcia, Spain; ffigueroa@ucam.edu (F.F.-M.); fgiron@ucam.edu (F.G.); jagabaldon@ucam.edu (J.A.G.)

**Keywords:** pulsed light, walnut, UV light, non-thermal, rancidity, sensory, volatiles, food quality, nut

## Abstract

Shelled walnuts are considered a microbiologically low-risk food but have been linked to some outbreaks, and a treatment aiming to decrease this risk is desirable. Pulsed light (PL) may be an alternative, providing it does not seriously impair their quality. This work assessed the impact of PL on some quality attributes of walnuts. To do this, measurements of rancidity, volatiles, total phenols, antioxidant activity, and descriptive sensory analysis were carried out on untreated and PL (43 J/cm^2^)-treated kernels. PL had no statistically significant (*p* > 0.05) effects on TBARS, peroxide value, total phenols, and antioxidant activity but significantly increased the concentration of volatiles related to green/herbaceous odors and decreased compounds related to fruity and citrus odors. The descriptors nut overall, walnut odor and flavor, and aftertaste were given statistically significantly (*p* < 0.05) higher scores, while descriptors woody odor and sweet received lower scores; 16 other traits such as all those related to color, texture, and rancidity were unaffected. No significant (*p* > 0.05) effects on total phenols and antioxidant activity in general were observed during the course of PL treatment. It can be concluded that PL technology may be used in shelled walnuts with only mild effects on their quality; a storage study must be carried out in order to determine the effect of PL treatment on its shelf-life.

## 1. Introduction

Pulsed light (PL) is a non-thermal processing technology based on the application of repetitive flashes of a non-coherent, broad-spectrum, high-intensity light. It has been developed in the context of the technologies aimed to increase the safety of foods avoiding the deleterious effect of heating. Its spectrum is composed of infrared, visible light, and UV light. The UV-C sub-band accounts for much of its microbicide action. Its application is limited to the decontamination of surfaces and UV-transparent liquids because of its low penetration power [1]. The capability of PL to yield fast effects is one of its main features. It is more efficient than low- and medium-pressure mercury lamps on a per time and fluence basis, and it is more ecologically friendly than them because lamps are filled with an inert (xenon) gas rather than mercury [2]. Fluence (J/cm^2^) is the unit used to characterize PL treatments; it is the measure of the amount of energy impinging a target surface per amount of target surface [3].

The efficacy of PL to inactivate microorganisms on food surfaces has been successfully tested in a wide variety of foods, including nuts such as almond kernels [4] and shelled walnuts [5].

Walnuts (*Juglans regia* L.) are considered a low-risk food from the microbiological point of view due to their low water activity; however, several outbreaks caused by their consumption have been reported. In 2011, an outbreak linked to the consumption of shelled walnuts contaminated by *Escherichia coli* O157:H7 was reported in Canada. Some affected people were hospitalized, and a few developed hemolytic uremic syndrome [6]. *Salmonella* has been detected in walnuts [7,8,9,10] and in a pre-packed mixture of nuts that included walnuts [11]. A total of 11 out of 20 walnut recalls due to food pathogen concerns registered in the USA between 2010 and 2015 were linked to *Salmonella* [12]. This pathogen has been shown to be capable of long-term survival on the surface of walnut kernels [8]. Santillana-Farakos et al. (2019) [13], based on a quantitative risk assessment of human salmonellosis from consumption of walnuts in the United States, have predicted that a minimum 3-log *Salmonella* reduction treatment would result in less than one case of salmonellosis per year linked to the consumption of walnuts. It has recently been shown that PL can achieve that level of *Salmonella* inactivation in shelled walnuts [5] when applying 41.2 J/cm^2^. However, this finding would not have a practical application if PL deteriorates the quality of nuts. Walnuts are very rich in lipids; they are rich in polyunsaturated fatty acids with a high linoleic acid content and particularly high ω3:ω6 ratio, which is the highest of all the tree nuts [14]. It is well known that the exposure to UV light in foods with high amounts of unsaturated fatty acids in the presence of oxygen promotes rancidity; therefore, a detailed analysis of the potentially harmful effects of PL applied to shelled walnuts at a fluence level required to reach 3-log *Salmonella* reduction is deserved. Izmirlioglu et al. (2020) [5] reported that PL has no significant effect on the color nor malondialdehyde levels (rancidity indicator) of shelled walnuts; however, the fluences applied to the samples used in these determinations were far below the fluence they used to achieve the 3-log *Salmonella* reduction. Furthermore, walnuts have a wide arrange of volatile compounds that affect their quality perception and stability, and the potential generation of new volatile compounds after walnut processing by novel technologies should be assessed.

Walnuts exhibit interesting nutritional properties due to their high content of linoleic acid and phenolic compounds, which have antioxidant activity [15,16]. The preservation of their antioxidant capacity is desirable not only because it is related to health properties but also because it helps to prevent fatty acid oxidation [17].

Therefore, in order to assess the actual potential application of PL to achieve 3-log *Salmonella* reduction in shelled walnuts without significantly harming their quality, a comprehensive evaluation of the effect of the required fluence (41.2 J/cm^2^) on the quality of this product is deserved. Research with this aim was undertaken, which included measurements of lipid oxidation, volatile compounds, and descriptive sensory analysis together with determinations of health-related compounds such as total phenols as well as antioxidant capacity.

## 2. Materials and Methods

### 2.1. Walnut Source and Composition

Walnuts (*Juglans regia* L. var. Chandler) were provided by a local supplier from Orihuela (Alicante, Spain) and stored at 8 °C until used. Walnut shells were mechanically broken and kernels extracted and halved.

Moisture was determined by drying the samples to constant weight at 95–100 °C (AOAC method 925.40, 2005) [18]. Ashes were quantified by means of sample incineration in a muffle furnace as described by AOAC [19]. The protein content in walnuts was calculated by multiplying the total nitrogen content obtained by the Kjeldahl method [20] by a conversion factor of 5.30 [21]. The quantitative determination of the total oil of the samples was carried out by extraction in Soxtec equipment (Avanti 2055) according to AOAC [22]. Carbohydrates were calculated by difference.

### 2.2. Walnut Treatment

PL was applied to shelled walnuts by using a commercial system (XeMaticA-Basic-1 L, Steribeam, Germany), whose characteristics can be found in Pérez-López et al. (2020) [23]. Eight halves of walnuts were placed in two rows along the longitudinal axis of the lamp below it. The position of halves was changed clockwise in order to maximize a homogeneous exposition to the lamp. Once the pre-set number of pulses was delivered, samples were turned upside down, and the same number of pulses was applied on the other side. Treatments were carried out in triplicate, and samples were analyzed immediately post treatment or their oil immediately extracted, depending on the intended analysis.

Light pulses were generated at 2.5 kV, which gives place to a characteristic emission spectrum that has previously been reported [24]. Each light pulse delivered a fluence at the walnut surface of 2.14 J/cm^2^. Samples were treated with 0, 5, 10, 15, or 20 pulses, which correspond respectively to fluences of 0, 10.7, 21.4, 32.1, and 42.8 J/cm^2^.

### 2.3. Lipid Oxidation

In order to extract the oil, the shell was removed from the samples manually until obtaining 25 g of walnut. This quantity was mixed with 250 mL of hexane (1:10 *w/v*) (Scharlau, Sentmenat, Spain) and homogenized in Ultraturrax T-18 basic for 3 min. The lipid fraction was taken to a rotary evaporator, distilling under vacuum until the removal of the hexane. The hexane-free oil was dried over sodium sulfate (Merck Darmstadt, Germany) and filtered (Whatman # 4 filter paper). The samples were stored in the dark at −80 °C until further analysis (up to 24 h).

#### 2.3.1. Thiobarbituric Acid Reactive Substances (TBARS)

For the determination of TBARS, 5 mL of oil and 5 mL of thiobarbituric acid solution (Sigma, Steinheim, Germany) were mixed and heated for pink color development at 80 °C for 40 min in a water bath. Once finished, samples were cooled to room temperature (~22 °C; 1 h), and the absorbance (532 nm) was measured with a Varian Cary 50 Bio spectrophotometer (Varian, Palo Alto, CA, USA). The concentration of TBARS in samples was obtained from a standard curve of malondialdehyde (Sigma, Steinheim, Germany) and expressed as mg of malondialdehyde per liter of oil [25].

#### 2.3.2. Peroxide Value

The method developed by Buege and Aust (1978) [26], with slight modifications, was used to the determination of the peroxide value (mmol O_2_/g of walnut oil). A total of 5 g of walnut oil and 30 mL of acetic acid solution (50% acetic acid (Normapur, NWR), 50% chloroform (Normapur, NWR)) were homogenized in a 250 mL test flask.

After vortexing, the samples were incubated in hot water (50 °C) for 30 min. Then, samples were filtered using Whatman # 4 filter paper. The filtrate was received in 0.5 mL of KI (Scharlau, Sentmenat, Spain) (50%), kept in darkness for 2 min, three drops of 1% starch (Panreac-Apllichem, Barcelona, Spain) were added (as an indicator), and the mixture was titrated with Na_2_S_2_O_3_ · 5H_2_O (Scharlau, Sentmenat, Spain) (0.01M) added dropwise until reaching the end point.

The peroxide value, expressed in milliequivalents of active oxygen per kg of oil, was calculated using the following formula: PV = volume of sodium thiosulphate × 0.1 N × 1000/mass of oil.

### 2.4. Volatile Compounds

The volatile composition of the walnut samples untreated (control) and subjected to a PL treatment of 42.8 J/cm^2^ was determined using headspace solid-phase micro-extraction (HS-SPME) following the method previously described by Noguera-Artiaga et al. (2020) [27]. Prior to the optimization of the method, tests were carried out to determine the amount of sample that offered the best signal results. After that, 2 g of sample, 1 g of NaCl, and 10 g of ultrapure water were weighed and added into a 20 mL vial. The sample was analyzed in a Shimadzu AOC-6000 Plus autosampler (Shimadzu Corporation, Kyoto, Japan), and after 10 min of equilibration time, a 50/30 μm DVB/CAR/PDMS fiber (1 cm) was exposed for 45 min at 40 °C to the sample headspace (throughout the extraction it was kept under constant agitation of 300 rpm). A GC2030 (Shimadzu Scientific Instruments, Inc., Columbia, MD, USA) was used for the separation of compounds, and a mass spectrometer detector (TQ8040 NX triple quadrupole mass spectrometer; Shimadzu Scientific Instruments, Inc., Columbia, MD, USA) was used to the detection. The chromatographic column used was Sapiens X5MS (Teknokroma, Barcelona, Spain), 30 m × 0.25 mm i.d., 0.25 μm film thickness. Only the single quadrupole acquisition mode was used on the TQ8040 NX (Q3 Scan; event time 0.100 s; mass range 40–300 *m/z*; scan speed 5000 amu/s). The oven temperature program was as follows:

(i) Initial temperature of 40 °C and hold for 5 min;
(ii) Ramp of 2 °C/min up to 140 °C;
(iii) Ramp of 5 °C/min up to 210 °C;
(iv) Ramp of 20 °C/min up to 230 °C, and hold 10 min.

The pressure at the head of the column was 50.4 kPa with a constant linear velocity mode of 36.3 cm/s. The interface temperature was 280 °C. The ion source temperature was 230 °C. The injector temperature was 250 °C. The carrier gas was helium, with a column flow of 1.01 mL/min, and the injector worked in splitless mode and with a purge flow of 6 mL/min.

A commercial alkane standard mixture (Sigma-Aldrich, Steinheim, Germany) was used to obtain the retention indexes, as well as the NIST 17 Mass Spectral and Retention Index Libraries. When it was based only on mass spectral data, the identification was considered tentative. Only compounds with spectra similarity >90% and with a deviation less than 10 units of linear retention similarity were considered as correct hits.

### 2.5. Descriptive Sensory Analysis

The sensory analysis of shelled walnuts untreated (control) and subjected to a PL treatment of 42.8 J/cm^2^ was performed by a panel of 10 highly trained panelists (aged 30 to 62 years; six female) from the Food Quality and Safety research group (Escuela Politécnica Superior de Orihuela, Spain). Each panelist had more than 1000 h of experience with sensory analysis of nuts and other foods. The methodology used for the descriptive sensory analysis was that previously described by Noguera-Artiaga et al. (2019) and Carbonell-Barrachina et al. (2015) [28,29]. The lexicon developed and used for the analysis of the samples is shown in Table 1. A total of 2 appearance descriptors were studied (color and color homogeneity), 6 odor descriptors (nut overall, walnut, roasted, woody, earthy, and rancy), 10 flavor descriptors (nut overall, walnut, roasted, woody, earthy, rancy, sweet, bitter, astringent, and aftertaste), and 5 texture descriptors (hardness, crunchiness, friability, adhesiveness, and oiliness). The scale used ranged from 0 to 10 (0 = without intensity and 10 = high intensity) with increments of 0.5 units. Ten walnuts were served to panelists in odor-free disposable 50 mL biodegradable cups, coded using 3-digit numbers at room temperature (~22 °C). To clean their palates between samples, unsalted crackers and mineral water were provided to panelists. Analyses were run in triplicate (*n* = 3).

### 2.6. Total Phenolic Compounds and Antioxidant Activity

Sample preparation was carried out following that described by Pérez-Jiménez et al. (2008) [30], with some modifications. Extracts were analyzed the same day of the treatments.

#### 2.6.1. Total Phenolic Compounds

For sample preparation, 5 g of walnuts were homogenized in an Ultraturrax T-18 basic for 2 min, at 24,000 rpm with 20 mL of a 3% methanol (Carlo Erba Reagents, France) + formic acid (Normapur, NWR) solution. The extracts were centrifuged for 10 min at 4000 x g in a Heraeus Biofuge Stratos centrifuge. The supernatant, methanolic extract, was separated from the precipitate and filtered through Whatman # 4 filter paper and collected in opaque flasks. The precipitate was subjected to a new extraction process under identical conditions, and its methanolic extract was joined to that obtained after the first extraction.

The content of total phenolic compounds (TPC) of walnut extracts was determined using the Folin–Ciocalteu method [31]. The reaction mixture contained 1 mL of walnut extracts, 5 mL of the Folin–Ciocalteu reagent (Merck; Darmstadt, Germany, and 20 mL of sodium carbonate (200 mg/L) (Scharlau, Sentmenat, Spain). The final volume was made up to 50 mL with distilled water. After 30 min of reaction, the absorbance at 765 nm was measured in a spectrophotometer Varian Cary 50 Bio (Varian, Palo Alto, CA, USA). The determinations were carried out in triplicate, and TPC content was expressed as g of gallic acid/L by using a calibration curve of pure gallic acid (Sigma-Aldrich, St. Louis, MO, USA).

#### 2.6.2. FRAP Assay

The analysis of antioxidant capacity by the ferric reducing antioxidant power (FRAP) assay was performed according to Benzie and Strain (1996) [32]. The determinations were carried out at 595 nm and 37 °C after 30 min of incubation in a spectrophotometer Molecular Devices LLC (Sunnyvale, CA, USA). Solutions of known Trolox (Sigma, Steinheim, Germany) concentrations are used for calibration. The antioxidant capacity was expressed as µM.

#### 2.6.3. DPPH Assay

The analysis of antioxidant capacity by DPPH radical scavenging was carried out according to Bondet et al. (1997) [33]. Briefly, 5 μL of each sample was added to 1 mL of 2,2-diphenyl-1-picrylhydrazyl (Sigma, Steinheim, Germany) solution (0.094 mM in methanol). Then, after 60 min at 20 °C, the absorbance of samples was determined at 515 nm in a spectrophotometer. The antioxidant capacity was reported as µM equivalents of Trolox.

#### 2.6.4. ABTS Assay

To determine the scavenging capacity of walnuts against the ABTS radical was performed the method previously described by Miller and Rice-Evans (1997) [34]. The blue-green ABTS^•+^ was produced through the reaction between 2,2′-azino-bis(3-ethylbenzothiazoline-6-sulfonic acid (Sigma, Steinheim, Germany) and activated manganese dioxide (Scharlau, Sentmenat, Spain) in water. Then, the solution was kept overnight in darkness before use. To obtain the results, 900 mL of ABTS^•+^ solution was added to the samples, and after 6 min of incubation in the darkness, at room temperature (22 °C), the absorbance was measured at 734 nm. Results were expressed as µM equivalents of Trolox.

### 2.7. Statistical Analysis

Data were analyzed for normality and homoscedasticity by the Kolmogorov–Smirnov and Levene tests, respectively, and for significant differences by one-way ANOVA and Tukey test, using IBM SPSS Statistics 27. Experiments were repeated three times.

## 3. Results and Discussion

The proximal composition of the walnuts used in this research was 3.60 ± 0.41 moisture, 16.26 ± 0.70 proteins, 65.73 ± 1.30 oil, 2.77 ± 0.55 ashes, and 11.65 ± 0.12 carbohydrates. These values are in harmony with previous reports [35].

### 3.1. Lipid Oxidation

It is widely known that the combination of unsaturated fatty acids, oxygen (from air), and UV light gives place to lipid oxidation, which results in rancidity [36]. All these players are present in the treatment of shelled walnuts by PL; therefore, the assessment of the potential sensory changes caused by PL at recommended microbicide fluences is warranted. Oil stability in walnuts is of paramount importance because it is a determinant of their shelf-life; indeed, considerable research has been performed to avoid lipid oxidation in walnuts during storage [37,38,39].

Table 2 shows the results of measurements of lipid oxidation in walnuts at increasing fluences. Both rancidity indicators, TBARS and peroxide, values were not significantly (*p* > 0.05) changed by PL treatment. Similarly, almonds subjected to a PL treatment (after 1 min water dipping) with the fluence required to achieve a 5-log reduction in *Salmonella* counts did not exhibit a significant increase in lipid oxidation even after 11 days of storage at 39 °C, and no differences in color and appearance were observed [40]. In comparison, the peroxide value of walnut kernels increases at gamma irradiation doses as low as 1.0 kGy [41], and an off-flavor related to oxidative rancidity has been reported for X-ray-treated shelled walnuts [42]. These results point toward a mild character of PL technology for shelled walnut processing. However, because oil stability is a critical attribute of shelled walnuts, more extensive evaluations were undertaken, which are reported next.

### 3.2. Volatile Compounds

Twenty volatile compounds were found in the volatile composition of walnuts (Table 3). It is necessary to emphasize that the nuts used in this study were not exposed to a roasting treatment. These treatments increase the content of volatile compounds in the nuts, so in this case, the content of volatile compounds found is significantly lower than in other studies.

The compounds found with the highest intensity were 1-hexanol, hexanal, methyl hexanoate, and limonene, although their quantity varied significantly among the treatments studied. These compounds also correspond to those found as the majority in walnut and walnut oil [43,44].

The control sample presented a higher amount of methyl hexanoate and limonene (45.55% and 18.20%, respectively), compounds related to fruity and citrus odors, according to the SAFC Flavors and Fragrances Catalog (SAFC, 2011) [45]. On the contrary, the sample subjected to PL treatment was mainly characterized by the presence of 1-hexanol and hexanal (27.83% and 30.11%, respectively), compounds related to green/herbaceous odors [46]. These four aromatic compounds are present in the volatile composition of this type of nut [37], although their concentration varies depending on the cultivar and agronomic conditions. In this case, the variation in its quantity is directly related to the treatment used. Hexanal has been used as a lipid oxidation marker [39]; however, it has also been identified as the second-most-important volatile compound of the Chandler walnut variety, with concentrations three- and six-fold higher than in the varieties Harley and Lara [43]. On the other side, Crowe et al. (2002) [47] identified hexanal as a walnut oil oxidation product but did not detect it in unoxidized walnuts, while this compound is considered a key important contributor to walnut kernel aroma [46,48]. These contradictory roles assigned to hexanal in Chandler walnut quality deserve to be further evaluated by sensory analysis.

Furthermore, statistically significant differences (*p* < 0.05) were also found in the relative content of ethyl hexanoate, limonene, nonanal, 4,6-dimethyl-dodecane, tetradecane, and isocaryophyllene. In all these compounds, the relative concentration was lower when the PL treatment was applied. These compounds are sensorially related to odors associated with fruity, citrus, and/or spices.

In general, according to the analysis of volatile compounds, the application of PL treatment to walnuts maintains the concentration of volatile compounds related to green nuts.

### 3.3. Descriptive Sensory Analysis

The descriptive analysis of the walnut samples had statistically significant differences (*p* < 0.05) in only 7 out of the 23 descriptors analyzed (Table 4). The application of light pulses in the walnuts did not have a significant effect on the appearance of the nuts. No differences were found in the color between the control samples and pulses samples (5.5 and 6.0, respectively) or in its color distribution around the inner nut (color homogeneity).

Differences were found in the odor of the samples (Table 4). The walnuts that were subjected to PL treatment appeared to have a more intense odor of nut overall (3.5) and walnut (3.5) and a lesser odor of woody (1.0) than the control samples (3.0, 3.0, and 1.5, respectively). The same results were found in the case of the taste analysis of the samples (Table 4). PL-treated walnuts had a slightly higher intensity of the sensory descriptors of overall walnut (8.5), walnut (8.0), and aftertaste (5.5) than the control samples (8.0, 7.5, and 5.0, respectively). However, the treatment slightly depleted the sweetness of the samples (2.5 control vs. 2.0 pulse). No rancidity was perceived by the panel, which is in harmony with the results shown in Table 2 and reinforces the conclusion about the lack of effect of PL technology on the stability of walnut oil.

Nevertheless, the increased hexanal content in PL-treated walnut kernels deserves further consideration. Previous studies on the oil stability of walnuts [49] and another nut (almond) [50] treated with PL have not included the determination of volatile compounds. However, the volatile composition after PL treatment of other foods less related to walnuts, namely, chicken fillets [51] and Manchego and Gouda cheeses [52], have also revealed an increase in hexanal content together with its lack of relevance to the sensory evaluation, which is in line with our result.

In the case of texture, no statistically significant differences (*p* > 0.05) were found in any of the parameters studied: hardness, crunchiness, friability, adhesiveness, and oiliness (Table 4).

After the sensory analysis of the samples, it can be concluded that the application of PL to walnuts slightly increases their intensity of characteristic odor and flavor without modifying their texture or appearance.

### 3.4. Total Phenolic Compounds and Antioxidant Activity

Phenolic compounds have been associated with benefits to health and have antioxidant activity. Walnuts have the highest known levels of phenolic antioxidants among all nut species [14]; as an important source of these compounds, their stability under treatment conditions is very important. Table 2 shows the concentration of total phenols and the antioxidant activity, measured by three different methods, of walnuts subjected to PL treatment. No significant (*p* > 0.05) effects on total phenols were observed during the course of PL treatment. As for antioxidant activity, no significant (*p* > 0.05) effects were observed except for a significant (*p* < 0.05) decrease in FRAP values, yet, overall, no effect on antioxidant activity can be concluded.

## 4. Conclusions

The effect of PL on oil stability, total phenols, antioxidant activity, volatile profile, and descriptive sensory quality of shelled walnuts was assessed under fluence levels relevant to microbiological safety (42.8 J/cm^2^). PL had no statistically significant (*p* > 0.05) effects on TBARS, peroxide value, total phenols, and antioxidant activity in general. PL significantly increased (*p* < 0.05) the concentration of volatiles related to green/herbaceous odors and significantly decreased compounds related to fruity and citrus odors. The descriptors nut overall and walnut odor and flavor and aftertaste were given statistically significant (*p* < 0.05) higher scores, while descriptors woody odor and sweet received statistically significant (*p* < 0.05) lower scores, and 16 other traits such as all those related to color and texture including the rancid trait were unaffected. No significant (*p* > 0.05) effects on total phenols and antioxidant activity in general were observed during the course of PL treatment. Therefore, it can be concluded that PL technology may be used in shelled walnuts with mild effects on their quality; a storage study must be carried out in order to determine the effect of PL treatment on the shelf-life of shelled walnuts.

## Figures and Tables

**Table 1:** Appearance, flavor, and texture attributes and definitions used in the study.

Sensory Descriptor	Definition	References and Intensities
Color	Visual evaluation of color intensity of sample	Pantone 17-1052 TCX = 8.5
Color homogeneity	Distribution of the main color in the sample	% of total of sample
**Odor and flavor**		
Nut overall	The nut-like aromatic that is typical of nuts such as pistachios and almonds.	Nuts Mix “Borges” = 8.0
Walnut	Aromatic reminiscent of walnut	NOW Foods Raw Walnuts = 8.0
Roasted	Dark-brown odor and flavor notes of products cooked without including bitter or burned notes	Roasted peanuts (Planters) = 5.0
Woody	Aroma associated with woody notes such as those associated with dried fruit shells	Whole peanuts (with shell) = 8.5
Earthy	Aroma related to wet dirt	Pomegranate “Mollar de Elche” = 5.0
Rancy	Aroma related to fat rancidity	Standard of the International Olive Council 5 g L^−^^1^ = 3.5
**Basic tastes**		
Sweet	The taste stimulated by substances such as sucrose or stevia	Sucrose solution 2.5 g L^−^^1^ = 3.5
Bitter	The taste stimulated by substances such as caffeine or quinine	Caffeine solution 1 g L^−^^1^ = 3.0
Astringent	The puckering or shrinking of the mouth caused by substances such as alum or tannins.	Alum solution 1.5 g L^−^^1^ = 1.5
Aftertaste	Time that the characteristic flavor of walnut remains in the mouth after swallowing or expectorating the sample.	5 s = 1.0 20 s = 10
**Texture**		
Hardness	The force required to bite food with molar teeth. Evaluate with the molars and on first bite.	Carrots = 7.5 Land O’Lakes American Cheese = 3.0
Crunchiness	Sound associated with mastication of sample with molars	Cheerios = 7.5
Friability	After chewing, the number of pieces the food breaks into sunke	Carrots fresh = 1.5 Soft brownie Little Debbie = 8.5
Adhesiveness	Amount of product that remains adhered to the teeth after chewing	Mushrooms unpeeled = 2.0 Chocolate MilkyWay bar = 9.5
Oiliness	Oily sensation left in the mouth after chewing the sample	Lay’s potato chips = 8.0

**Table 2:** Quality indicators of shelled walnut oil subjected to pulsed light treatment (mean ± standard deviation).

	Fluence (J/cm^2^)				
	0	10.7	21.4	32.1	42.8
**Rancidity indicators**					
TBARS ^1^ (mg MDA ^2^/L oil)	0.27 ± 0.07 a	0.32 ± 0.06 a	0.27 ± 0.08 a	0.31 ± 0.07 a	0.34 ± 0.03 a
PV ^3^ (mmol O_2_/g oil)	9.13 ± 0.46 a	9.13 ± 0.64 a	9.27 ± 0.46 a	8.60 ± 0.35 a	8.73 ± 0.23 a
**Phenols**					
Total phenols (g GAE ^4^/l)	0.70 ± 0.17 a	0.69 ± 0.16 a	0.67 ± 0.08 a	0.60 ± 0.07 a	0.67 ± 0.14 a
**Antioxidant capacity**					
FRAP ^5^ (µM Fe^2+^ equiv.)	753 ± 55 a	531 ± 43 b	457 ± 21 b	674 ± 15 a	495 ± 13 b
DPPH (µM equiv. Trolox)	469 ± 32 a	464 ± 31 a	466 ± 29 a	469 ± 24 a	462 ± 22 a
ABTS (µM equiv. Trolox)	399 ± 30 a	396 ± 57 a	327 ± 36 a	391 ± 45 a	336 ± 79 a

^1^ Thiobarbituric acid reactive substances, ^2^ malondialdehyde, ^3^ peroxide value, ^4^ gallic acid equivalent, ^5^ ferric reducing antioxidant power. Within rows, values followed by different letters are statistically different (*p* < 0.05).

**Table 3:** Volatile composition (% relative area) and odor descriptors of walnut organic volatile compounds subjected or not to pulsed light (PL) treatment.

RT (min)	Compound	Odor Description	KI Exp.	KI Lit	ANOVA	Control	PL
7.199	Hexanal	Green, woody, grassy	800	801	***	3.02 b	30.11 a
10.968	1-Hexanol	Herbaceous, green	862	865	***	4.43 b	57.83 a
11.754	Methyl hexanoate	Fruity	915	915	***	45.55 a	0.20 b
16.079	Methyl-2-hexenoate	Fatty	934	933	NS	1.02	0.08
16.775	Benzaldehyde	Bitter almond, cherry, nutty	936	935	NS	1.85	1.11
17.846	1-Heptanol	Musty, leafy, herbal, green, sweet	970	970	NS	0.80	0.48
18.712	Methyl heptenone	Citrus, green	992	994	NS	0.98	1.41
19.803	Ethyl hexanoate	Fruity	1002	1000	**	5.81 a	0.34 b
21.439	β-Cymene	Terpenic	1025	1028	NS	1.82	0.38
21.790	D-Limonene	Citrus, orange, lemon	1029	1031	***	18.20 a	1.80 b
23.982	Sabinene hydrate	Herbal, cooling	1101	1096	NS	0.85	1.04
27.601	Nonanal	Waxy, citrus, green	1115	1105	***	3.10 a	0.95 b
31.633	(*E*)-Pinocarveol	Herbal, woody, pine	1145	1140	NS	0.93	0.75
34.432	Ethyl octanoate	Waxy, sweet, fruity	1201	1206	NS	0.73	0.20
34.760	Dodecane	-	1206	1200	NS	1.84	0.96
35.007	Decanal	Sweet, waxy, orange	1210	1207	NS	1.84	0.73
35.681	Tridecane	-	1298	1300	NS	0.50	0.24
40.031	Dodecane, 4,6-dimethyl-	-	1327	1325	**	3.06 a	0.84 b
48.395	Tetradecane	-	1401	1400	**	1.55 a	0.44 b
49.082	Isocaryophyllene	Woody, spicy	1459	1461	**	2.22 a	0.13 b

KI exp.: Kovat’s index experimental; KI Lit.: Kovats index literature (NIST). Values (mean of 3 replications) followed by the same letter within the same volatile compound were not significantly different (*p* > 0.05), according to Tukey’s least significant difference test. NS = not significant at *p* > 0.05; **, ***, significant at *p* < 0.01 and 0.001, respectively.

**Table 4:** Descriptive sensory analysis of walnuts affected by pulsed light (PL) treatment.

Sensory Descriptor	ANOVA	Control	PL
**Appearance**			
Color	NS	5.5	6.0
Color homogeneity	NS	8.0	8.0
**Odor**			
Nut overall	**	3.0 b	3.5 a
Walnut	**	3.0 b	3.5 a
Roasted	NS	0.5	0.5
Woody	***	1.5 a	1.0 b
Earthy	NS	0.5	0.5
Rancy	NS	0	0
**Flavor**			
Nut overall	**	8.0 b	8.5 a
Walnut	***	7.5 b	8.0 a
Roasted	NS	2.2	2.5
Woody	NS	3.5	4.0
Earthy	NS	1.5	1.5
Rancy	NS	0	0
Sweet	**	2.5 a	2.0 b
Bitter	NS	3.5	3.5
Astringent	NS	2.5	2.0
Aftertaste	**	5.0 b	5.5 a
**Texture**			
Hardness	NS	5.5	5.5
Crunchiness	NS	6.5	6.5
Friability	NS	7.5	7.0
Adhesiveness	NS	6.0	6.5
Oiliness	NS	2.5	2.5

Values (mean of 3 replications) followed by the same letter within the same volatile compound were not significantly different (*p* > 0.05), according to Tukey’s least significant difference test. NS = not significant at *p* > 0.05; **, ***, significant at *p* < 0.01 and 0.001, respectively.

## Data Availability

Not applicable.
